# Geographical Network Analysis

**DOI:** 10.1111/tesg.12480

**Published:** 2021-08-04

**Authors:** Justus Uitermark, Michiel van Meeteren

**Affiliations:** ^1^ Department of Geography, Planning, and International Development Studies University of Amsterdam Amsterdam the Netherlands; ^2^ School of Social Sciences and Humanities Loughborough University Loughborough UK

**Keywords:** Social network analysis, geographical network analysis, complexity theory, computational social science, history of geography

## Abstract

As the volume of digital data is growing exponentially and computational methods are advancing rapidly, network analysis is an increasingly important analytical tool to understand social life. This paper revisits the rich history of network analysis in geography and uses insights from that history to review contemporary computational social science. Based on that analysis, we synthesize the distinctive qualities of what we term geographical network analysis. Geographical network analysis presumes that networks are situated, construed through meaning, and reflect power relations. Instead of pursuing parsimonious explanations or universal theories, geographical network analysis strives to understand how uneven networks develop across space and within place through a constant back and forth between abstraction and contextualization. Drawing on the articles in this special issue, this paper illustrates how geographical network analysis can be put to work.

## INTRODUCTION

In the early days of the COVID‐19 pandemic, *The New York Times* journalist Benedict Carey profiled the Network Science Institute at Northeastern University (Carey 2020). Inside, physicists and computer scientists were piecing together data, from census data to social media networks, to forecast where the virus would break out next. The effort was bolstered by producing impressive maps and network diagrams to show where the epidemic might be heading. The real‐time modelling of infectious diseases is just one example of spatial scientific work that has been enabled by the explosive growth of digital data and the rapid advancement of computational methods. The Network Science Institute is one of a large number of centres that apply techniques and methods developed within the natural sciences to study the geographies of social life.

Work in this emergent field of computational social science is often underpinned by a specific ontology and epistemology where the objects of social scientific analysis are viewed as akin to the objects of natural or biological analysis (Barnes & Wilson 2014; Törnberg & Törnberg 2018). Such epistemic assimilation takes place through the language of ‘complexity’, which has become the *lingua franca* for computational social science.^1^ Virtually all phenomena – ranging from brains and ecosystems to economies and language – can be analysed as ‘complex systems’ whose defining quality is that higher‐order structures emerge out of microlevel interactions. Computational methods and digital data have opened up exciting new opportunities for research across the social sciences (Watts 2013, 2014) and promise to produce fresh insights for geography (O’Sullivan & Manson 2015). Within this broad field, ‘geocomputation’, ‘spatial network analysis’, and ‘geographic data science’ explicitly examine spatial questions (Couclelis 1998; Barthélemy 2011; Harris *et al*. 2017; Singleton & Arribas‐Bel 2021).

Although the burgeoning field of computational social science advances both spatial and network methods, much of this work takes place outside of the disciplinary boundaries of geography, affirming Couclelis' (1998, p. 42) contention that ‘not all geography is spatial science and not everything spatial may be interpreted geographically’. If the geographical analysis of networks is not the same as spatial network analysis, then what is it? As physicists and computer scientists are exploring spatial questions, what defines a distinctly geographical approach to network analysis?

We do not have to start from scratch to answer these questions. Geographers have a rich history of network analysis that must be recollected, reassessed and reinvented in the contemporary age where network analysis has become ubiquitous in academic research and, indeed, in social practice at large. When network analysis was first introduced in geography in the 1950s and 1960s, it was part of a broader movement to redefine geography as ‘spatial science’ (Bunge 1962 [1966]). As radical and humanist geographers entered the scene in the 1970s, they critiqued this spatial science perspective, drawing on other (ecological, historical) strains of geographical thought (Buttimer 1974). One of the main arguments advanced in these debates is that the spatial science perspective relied on a reductionist understanding of geography and space (Barnes & Wilson 2014; Derudder & Van Meeteren 2019; Poorthuis & Zook 2020). Since that time, geography evolved in a pluralist discipline where radically different approaches coexist (Cox 2014). Depending on who you ask, human geography became a discipline where these approaches sit isolated side by side (Johnston 2020) or facilitate a pluralist cross‐fertilization between traditions (Holt‐Jensen 2018). In this special issue, we aim for the latter and explore how network analysis can engender conversations and cross‐fertilization among different lines of geographical practice.

Learning from the history of network analysis within their discipline, geographers could play an important role in challenging and amending the problematic ontological and epistemological assumptions inherent in computational social science and complexity science. Complexity science, owing to its roots in the natural sciences, has had difficulty grasping with qualitative change, meaning and top‐down causality (Couldry & Hepp 2017; Törnberg 2017; Törnberg & Törnberg 2018; Andersson *et al*. 2019). Examining social life – including spatial processes such as segregation, migration and gentrification – through the prism of complexity often naturalizes and thus obscures underlying power dynamics (Uitermark 2015). Moreover, Kitchin (2014, p. 8) argues that computational social science is ‘sacrificing complexity, specificity, context, depth and critique for scale, breadth, automation and descriptive patterns’, whereas Franklin (2021, p. 55) laments that newly emerging fields in spatial and computational analysis, like ‘urban science’ or ‘urban data science,’ are ‘almost completely divorced from substantive geographical domain expertise’.

Geographers are in a good position to address these issues as they have a rich history of considering how a range of forces act in concert and in context in particular cases. Although complexity science is geared to extract and abstract, geographers are pre‐disposed to contextualize and specify (see also O’Sullivan & Manson 2015). There are not only substantive but also strategic and pragmatic reasons for such a venture. Geographers have begun experimenting with computational social science methods to re‐evaluate old geographical theories, for instance, on information diffusion (Peris *et al*. 2021), central place theory (Van Meeteren & Poorthuis 2018) and the potential of the approach is flagged for whole subfields such as urban geography (Grekousis 2019) or health geography (Davies & Green 2018). Notwithstanding these studies being part of a growing movement of renewed quantitative and computational geography in certain niches of the discipline (Batty 2013, 2017; Arribas‐Bel & Reades 2018; Poorthuis & Zook 2020; Wolf *et al*. 2020; Franklin 2021; Singleton & Arribas‐Bel 2021), most geographers today are not specialists in computational methods. This is important to acknowledge because it implies that computation and modelling in the pursuit of universal mechanisms and patterns will not be the bread and butter of geography even if we would believe that the social world lends itself to such a pursuit. If geography is an ‘interdisciplinary discipline’ (Baerwald 2010), a specific way of synthetic thinking whose strength is bringing different strands of knowledge and methods to bear on the world (Harris 1971; Holt‐Jensen 2018), the question becomes how to incorporate network analysis into broader research strategies to study how a range of forces act in concert and in context.

Geographical network analysis is the general label we use for this endeavour. In the remainder of this paper, we first attend to historical precedents of network analysis in geography, revisiting some of the early claims and critiques. We then outline in broad contours the distinctive qualities of geographical network analysis before explaining how the contributions to this special issue exemplify different ways of doing geographical network analysis.

## PIONEERS OF NETWORK ANALYSIS IN GEOGRAPHY

In his historical account of social network analysis, Linton Freeman (2004) considers the period between 1940 and 1970 as social network analysis' ‘dark ages’. Sociometric studies of communities and small groups had flourished in the 1930s (Loomis & Pepinsky 1948), but after the Second World War, American sociology becomes infatuated with survey methods and neglects the intermediate scales between the ‘individual’ and ‘society’ (Abott & Sparrow 2007). Remarkably enough, quantitative geographers become the torchbearers of social network analysis in these dark ages (Freeman 2004). Why did geographers embrace an approach that had been cast aside by sociologists a decade earlier?

The vanguard in American geographical thinking in the 1950s wanted to move away from studying static and bounded regions to examining flows and interactions (Ullman 1980 [1954]). This focus on dynamism contrasted sharply with sociologists' interest in immutable social structures and putatively static nation‐states. In Nelson's (2019) terms, geographers developed from thinking of the world in terms of mosaics to thinking in terms of tapestries, whereas sociologists moved in the opposite direction. Researching dynamic regions quantitively required data and computational power, and the pioneer geographers discussed by Freeman (2004) addressed this issue head on. Swedish geographer Torsten Hägerstrand's dissertation on spatial diffusion (1967 [1953], p. 167) uses simulation methods to scale up small‐scale sociometric insights into probability estimations of large‐scale population movements. William Garrison, a quantitatively minded transport and economic geographer, introduced network analysis to his group of eager young graduates at the University of Washington in Seattle, USA, including Brian Berry, Michael Dacey, Duane Marble, Richard Morrill, Bill Bunge and John Nystuen (Barnes 2004). Garrison and his group borrowed graph theoretical approaches from operations research (Garrison 1960a) and became enchanted by the prospect of using network analysis to optimize transport systems (Garrison 1960b). Garrison's group adopted mainframe computers as soon as they became available to perform the matrix algebra underlying network analysis (Barnes 2004; Freeman 2004, p. 83). Computational applications, fuelled by their Cold War Era demand in operations research (Barnes 2004; Barnes & Wilson 2014), thus allowed geographers to operationalize and test relational theories they had been developing (Van Meeteren 2019).

Hägerstrand was invited for a visiting scholarship in Seattle in 1959, and his interactions with Garrison's research group contributed significantly to geography's quantitative revolution (Barnes 2004). Network analysis plays a key role in Bill Bunge's *Theoretical Geography* (1966 [1962]) and Peter Haggett’s *Locational Analysis in Geography* (1965) that brought the quantitative revolution to the United Kingdom, where it cross‐fertilized with the ideas of physical geographer Richard Chorley. Studies of this period span subdisciplinary divides and examine highway networks (Garrison 1960b), citation networks (Bunge 1961), city networks (Nystuen & Decey 1961), historical geographical networks (Pitts 1965) and commodity flows (Berry 1966)^2^ with a similar epistemological frame. Network analysis at this point in time provided a shared vocabulary for geographers working on very different topics, promising to highlight patterns and mechanisms that operate across different fields.

In the context of the quantitative revolution, network analysis was more than just a novel approach. Burton (1963, p. 159) explicitly singles it out as a promising exemplar what theoretical geography could be. The New Geography would be a ‘science of space’, emphasizing ‘the spatial point of view’ where geography was defined by geometrical descriptions of spatial phenomena (Bunge 1966 [1962]; Taaffe 1974). Network analysis, cleanly described by a pure spatial‐mathematical language of topology, spoke to this agenda directly (Bunge 1966 [1962]). The pursuit of a universal vocabulary fit the technocratic optimism of the 1960s and, more prosaically, allowed geography to tap into the ‘hard science’ funding streams of the Cold War (Barnes 2004).

By abstracting reality into graphs with similar properties, phenomena as divergent as rivers, city systems, transport systems and flows of information could be meaningfully compared (Haggett & Chorley 1969). Graph theoretical thinking allowed to make simple models of complex structures (Haggett & Chorley 1967). Philosophically, the 1960s spatial science conceived of a network as a real existing object (a concrete transportation network, a river system, a city system), which you would abstract using graph theory (Haggett & Chorley 1969). These graphs were conceptualized with a Cartesian ontology that regarded the network as a self‐evident totality (Harvey 1995). Proponents of network analysis in geography acknowledged from the outset that their network abstractions were reductionist (e.g. Haggett 1965, p. 19). However, simplicity was heralded as a good thing. Network analysis was fascinating because it allowed filtering out signals within the noise (Gould 1979) to uncover the ‘hidden spatial order’ (Haggett 1965, p. 2; Bunge 1966 [1962]).

## CRITIQUES OF THE PIONEERS OF NETWORK ANALYSIS

This ‘hidden order’ of static geometrical patterns became contentious in the countermovement against spatial science in the 1970s. With hindsight, even many quantitative geographers considered the focus on geometry, and hence on network analysis, a weakness of early quantitative geography (Bennett & Wrigley 1981). By elevating geometry as the vocabulary of geography, other kinds of geography (humanist geography, cultural geography) were effectively relegated to backwaters of the discipline. As the dream of a rationally organized modernist society started falling apart in the 1970s, pleas by humanist and cultural geographers for a richer understanding of motives, context, and history gained ground. These scholars argued that a discipline only bound together by a shared set of methods and techniques would be doomed if it was not rooted in an emancipatory intellectual project (Harris 1971; Buttimer 1974). Successful spatial science methods would otherwise simply be opportunistically absorbed in other disciplines. The most talented practitioners would join more fertile disciplinary pastures elsewhere and leave the geography discipline at risk of being ‘dismembered’ or disappearing altogether (Harris 1971, p. 158). Joining the chorus of critique were radical geographers. From a radical or critical geography perspective, privileging the ‘hidden spatial order’ as an object of study affirms the societal status quo (Sheppard 1995). That ‘hidden order’ is not some foundational truth waiting to be discovered but the product of social relations (Couclelis 1983; Hanson 1983) and examines as the provisional outcome of power struggles (Massey 1995 [1984], 1993). Moreover, the search for orderly patterns led to confirmation bias and chasing self‐fulfilling prophecies in geographical practice (Szymanski & Agnew 1981). Sack (1972) extended the critique by showing that a narrow focus on geometry was insufficient for explanation. It is possible to use graph theory to compare river networks to city networks, but it cannot be assumed that inferences about network structures will carry over between these substantive domains. Assumptions derived from isomorphisms in topological structure from divergent networks are at best useful to generate hypotheses about common mechanisms of causation, but explanation will always require testing with domain knowledge (Verweijen & Van Meeteren 2015; Van Meeteren *et al*. 2016). Radical geographers introduced the notion of ‘spatial fetishism’ (Anderson 1973) to frame these critiques. Pursuing the discovery of ‘hidden spatial order’ paradoxically obscures the social forces shaping the status quo.

As we noted above, some of these critiques had been anticipated by proponents of spatial science. From the very beginning of the quantitative revolution, a number of geographers were aware that if you believe the world changes historically through relations and processes, focusing on patterns alone is reductionist (Blaut 1961). A more plastic conception of space in which the constitution of objects is taken into account is necessary to understand change (Blaut 1962). The more geographers thought and talked about ontologies of flow and movement (Blaut 1962; Harvey 1995), the more it became clear that a relational perspective of space, which has attention for the internal relations constituting objects, is imperative (Harvey 1995). Given these insights, by the late 1960s, it was clear to even the most mathematically inclined geographers that making inferences about processes using cross‐sectional patterns alone was impossible (Olsson 2016 [1968]; cf. Massey 1995 [1984]). As a result, the quantitative momentum came to a halt. Many of its pioneers abandoned positivism and adopted neo‐Marxist perspectives (Barnes 2004). Not only did the share of quantitative work within geography decrease, the share of geometry and network analysis within quantitative geography diminished too (Bennett & Wrigley 1981) as much quantitative talent went into the development of GIS (O’Sullivan in Harris *et al*. 2017, p. 601; Franklin 2021).

By the time social network analysis emerges from its hibernation in the late 1970s (Freeman 2004), little more than the incidental geographer (Pitts 1978) is still on the forefront on what is to become modern social network analysis. Neither do geographers play a significant role in the community of physicists that discover network analysis to study social phenomena in the late 1990s (Freeman 2011). Geography's network analysis tradition did not completely vanish – some worked in transportation and urban networks or regional science (e.g. Kipnis 1985; Thomas *et al*. 2003); others staunchly defended the innovations in quantitative geography of the 1960s (Gould 1997); yet, others worked beyond Anglophone academia (e.g. Cattan 1990; Dematteis 1997; Ducruet & Beaugitte 2014) – but it became marginal within the discipline in the 1980s and 1990s. Even as ‘networks’ become a central organizing metaphor in the 1990s to understand globalization (Castells 2002), it takes until the 2000s for geographers to adopt network analytical methodologies to empirically substantiate these network theories (Derudder 2021, this issue; Poorthuis & Van Meeteren 2021, this issue).

The spatial science advocated by the proponents of the 1960s' quantitative revolution in geography is in many ways similar to today's complexity science: they both eschew disciplinary boundaries, offer a common vocabulary to study a wide range of phenomena, focus on the rigorous description of regularities by means of mathematics, and aspire to construct elegant models with predictive power. Moreover, the anxiety within some quarters of geography about missing the boat in the current moment of big data and complexity is tangible, especially now that computational scholars are tackling geographical questions (Derudder & Van Meeteren 2019; Singleton & Arribas‐Bel 2021). The question whether the discipline needs to again rally around a method‐based definition, what Cupples (2020) terms ‘*geoscientization*’, is never far off, particularly when prosaic interests such as the discipline's grant capturing capacity are debated or in which faculty geographers ought to be located. Considering these similarities, this history of network analysis in geography offers important lessons for contemporary debates. Many of the critiques articulated against the spatial science in the 1960s hold *mutatis mutandis* for contemporary complexity science. Perhaps another lesson to be learned is that there is a risk of foreclosing opportunities for integrating different kinds of methods and perspectives. In the 1960s, as today, deep ontological and epistemological divides might give the impression that scholars face a fundamental and binary choice: either believe in the prospect of a monist universal scientific framework and adopt network analysis as a means to get there or reject universalism as a pipedream and discard network analysis. The debate among geographers in the 1960s and 1970s was most productive when participants acknowledged that it is possible to pragmatically use network analysis and other quantitative techniques without necessarily subscribing to the belief that such methodology provides the means to uncover an unambiguous hidden order or invariant laws (Wyly 2009).

## EPISTEMOLOGICAL PRECEPTS OF GEOGRAPHICAL NETWORK ANALYSIS

After a lull in network studies, there are omens of a revival (see also Derudder & Neal 2018; Radil & Walther 2018; Neal *et al*. 2021). Network analysis has been systematically incorporated into geographical research programs on city networks (e.g. Capello 2000; Glückler & Panitz 2021; Taylor & Derudder 2016; Neal *et al*. 2021) and innovation networks (Morrison 2008; Broekel *et al*. 2014; Glückler & Doreian 2016). A number of scattered, yet suggestive, studies highlight the possibilities for applying network analysis to a broad range of topics, including geopolitical conflicts (Flint *et al*. 2009), crime (Radil *et al*. 2010; Andris *et al*. 2021), economic agglomerations (Van Meeteren *et al*. 2016), urban subcultures (Boy & Uitermark 2017, 2020) and social movements (Van Haperen *et al*. 2018). This special issue seeks to build on this momentum and sketch in broad contours what the distinctive contribution of geographical network analysis might be.

In the present conjuncture, this means that geographers have to define their position and argue their relevance in relation to computational social science and the ontologies of complexity underlying much of the work within this interdisciplinary field. Ontologies centred on the notion of complexity capture some important qualities of social life: many, if not all, social norms, institutions and structures are indeed emergent in the sense that do not originate from a plan or design but are the unintended outcome of interactions among a plurality of actors (Lambooy 2002). However, there are some fundamental differences between complex structures produced by fish, ants and bees on the one hand and humans on the other – differences that are important to articulate what sets apart geographical network analysis from both the spatial science of the 1970s and computational social science today.

The first key difference is that, unlike bees or other insects, humans pursue collective goals consciously. ‘What distinguishes the worst of architects from the best of bees’, Marx argued, is that ‘the architect raises his structure in imagination before he erects it in reality’ (cited in Harvey 2000, p. 200). This already hints at a second important difference: humans make sense of social reality and act on meaning. There is a sharp difference here with the actors in ‘complex systems’ who typically are simple‐minded: they only observe their immediate environment, optimize on a single dimension and have a limited number of choices available. Assuming such actors can be helpful as a thought experiment – with Schelling's (1971) model of segregation as a paradigmatic example – but this is because it brackets just about everything that defines social life. A third difference follows from the first two: humans struggle with each other as they shape their environments according to their own ideas and interests (Uitermark 2015, 2017). A complexity perspective informs us that the structure we describe using network analysis has emergent properties (Sayer 2000, pp. 12‐13). Yet, because it is a social structure, and its constituents are human beings with degrees of freedom, making statements beyond probabilities on the evolution of the network structure is fraught with contention (Gould 1987). Any regularity is open to interpretation and contestation, imposing limits on the potential for parsimonious explanation in the social sciences.

All this is important for geographers because it casts the discipline in a different light: the analysis of particularities and variations not only is the incidental outcome of a specific disciplinary trajectory but also reflects the complexity of its object of study. ‘Complexity’ here does not just mean that higher‐order structures originate from lower‐level interactions but also refers to the multidimensional, variable, mediated and contested nature of social life. This has profound implications for the use of social network analysis. Whereas the rapidly expanding body of work on network analysis is dominated by physicists and sociologists searching for universal mechanisms and patterns, geographers are well positioned to analyse and explain variations, anomalies and particularities. Geographers can only hope to make distinctive contributions if they do not attempt to emulate the natural sciences but instead mobilize their specific disciplinary strengths, including domain knowledge, theoretical understanding of complex spatialities, an appreciation of particularities, and a commitment to mixed methods.

In consideration of geographers' historical engagement with network analysis and contemporary developments within computational social science, we tentatively identify six precepts of geographical network analysis.

First, geographical network analysis *takes into account different dimensions of space*. The fundamental difference between *spatial* network analysis and *geographical network analysis* is that the former tends to presume an absolute notion of space to which the network is grafted on where the latter does not. In line with its roots in the natural sciences, most analyses into spatial networks presume a Cartesian grid with fixed distances between its various co‐ordinates. Networks may enhance the interaction between co‐ordinates, indeed changing ‘relative distance’, the production of the co‐ordinates is taken for granted (Harvey 1995). The construction of a two‐dimensional grid or matrix that assumes the existence of ‘nodes’ and ‘edges’ as real co‐ordinates is a requirement for spatial network analysis. In addition, it is from this reduction that network analysis' appeal originates as it allows the researcher to highlight with mathematical precision one salient aspect of a messy reality. But geographers are well positioned to acknowledge the trade‐off associated with such abstraction as they have done much to develop conceptualizations of space that complement and transcend Cartesian understandings, emphasizing that space is not an empty container to be filled but produced through social practice (Lefebvre [1974] 1991), and develop ways to imagine and visualize these alternative conceptions (Bergmann & Lally 2021; Poorthuis & Zook 2020). Space and the entities populating it do not precede relations but are constituted through them (Massey 1993; Emirbayer 1997). The challenge is then twofold. First, to provide relational operationalizations of established notions like ‘region’ or ‘city’ (Bergmann & O’Sullivan 2018) and, second, to *situate* network analysis, acknowledging that it can only ever bring out one salient aspect of a place or space that is by definition unbounded, variable and multidimensional.

Second, it follows that geographical network analysis should *incorporate network analysis into holistic approaches*. The purpose of network analysis is to first extract from a particular context one salient dimension for closer inspection and then, in turn, contextualize the outcome. Casting an object in the topological language and visualization of network analysis is an act of representation (Harvey 1969). Whether the resulting abstract structures are an adequate representation to provide meaningful insights in developments in society cannot be taken for granted (O’Sullivan 2004). Geographical network analysis is constant back and forth between context and abstraction. Although computational social scientists and complexity researchers, just like the proponents of spatial science in the 1960s, examine a wide range of networks in pursuit of universal features (like power law distributions) or mechanisms (like preferential attachment) (Freeman 2011), abstracting away from context and content, geographers are pre‐disposed to depart from domain knowledge, allowing them to analyse networks in relation to the specific domain or place from which they were extracted.

Third, the process of extracting data and situating networks is predicated on the assumption that *all data are situated and* *performative* (Marres 2017). Computational social scientists tend to consider network data as ‘trace data’ or ‘data shadows’ (Shelton *et al*. 2014) that people unwittingly leave behind as they go about their life and work. As with spatial representation in maps (Harvey 1972; Wood 2010), network diagrams reduce reality to those aspects that the network visualiser deems important or theoretically relevant. Consequently, geographical network analysis assumes that all data are produced for specific purposes and under specific conditions. For instance, a connection between two Facebook users is not simply a ‘trace’ but a performative expression of a relationship. The study of ethnic segregation, to take another example, is at risk of falling prey to spatial fetishism if it takes ethnic and spatial categories for granted, implying the need for interpretative, historical and geographical analysis into the mutual constitution of space and ethnicity. Understanding networks thus requires an understanding of the production of the categories and data that underly them. This conception of data further implies that the goal of enquiry is not prediction but understanding or explanation (Sayer 1979; cf. Watts 2014).

Fourth, the networks in geographical network analysis *carry meaning and reflect power relations*. This means that we must take into account how social actors might have very different interpretations of what is registered as a ‘tie’. Interpretative work is essential because interpretations is inextricably a part of social reality: families, corporations or communities are not naturally occurring entities but are brought into being through the ascription of meaning. Relatedly, social networks of whatever kind are shot through with power relations – they do not express invariant laws or emerge organically from micro‐interactions but are the provisional and contingent outcome of co‐operation and competition between actors with different and occasionally conflicting ideas and interests. The dynamics of network formation play out within broad social structures, and the understanding the former, thus, requires an understanding of the latter.

Fifth, geographical network analysis' emphasis on multidimensionality, contingency and context provides an epistemological rationale for using a *mixed methods research* strategy. The key assumption here is that the various forces that interact in geographical space will be multidimensional by definition and can, therefore, not be grasped with any one technique or method. Whereas one rationale for mixed methods is triangulation (looking at the same thing from different perspectives as a means of validation), another rationale is to bring out different dimensions and factors. A commitment to mixed methods, thus understood, is not an expression of eclecticism but of a conviction that approaches that disregard either the materialist or phenomenological dimensions of social life are incomplete.

Sixth, geographical network analysis sees the use of network analytical methods as an opportunity for syntheses or conversations between subdisciplines. Network analysis provides a common language to bridge heterogenous knowledges across the thematic specializations and disciplinary silos that characterize contemporary geography. This does not mean that individual projects must take every factor into account, but it does mean that geography as a discipline should strive to add (not necessarily: accumulate) insights from a range of subfields on different patterns, dimensions and factors.

## EXAMPLES OF GEOGRAPHICAL NETWORK ANALYSIS: AN OVERVIEW OF THE SPECIAL ISSUE

This special issue brings together contributions from a range of subfields in geography to illustrate the potential of geographical network analysis. The seven full‐length papers bring these subfields in dialogue by applying the tools and vocabularies of network analysis within a variety of contexts. Each of the studies touches on the six dimensions of geographical network analysis to a certain degree although the emphasis varies from paper to paper.

Let us highlight two ways in which the contributions to this special issue illustrate how geographical network analysis reflects the engagement between geography and computational social science. First, we want to call attention to how the authors in this special issue use computational methods. The most salient computational method is the use of community detection algorithms to identify cohesive substructures within broader networks. Although Nelson (2020 [this issue]) elaborates the method in some detail, we here want to highlight how the method is incorporated into geographical analysis. Community detection is a salient example of the kind of ‘hidden order’ that computational methods can inductively derive from large data sets. Moreover, it has by now become so incorporated in standard network analytical software, that a non‐computational specialist has access to these algorithms at the push of a button. From the perspective of geographical network analysis, identifying these communities is not the end point of a research endeavour, but rather the creation of an explanandum: a structure in the data, which begs theoretical explanation and empirical exploration. Nelson (2020 [this issue]) uses community detection to identify regions but, importantly, embeds his analysis in long‐standing debates in geography on how to conceptualize regions. As a result, he does not treat the regions identified through computational methods as static or closed but as dynamic and porous. Similarly, Poorthuis and Van Meeteren (2021 [this issue]) emphasize that having clear theoretical expectations is key to resolve methodological judgment calls and disentangling the various causal mechanisms that collectively structure geographical networks. In De Craene *et al*. (2021 [this issue]) community detection reveals the temporal structure of subsequent generations of scholars involved in a Dutch geography magazine. This structure is then used to examine and narrate the history emerging from an otherwise unwieldy archive. Gamsu and Donnelly (2020 [this issue]) provide an example of how domain knowledge, qualitative methods and theoretical notions help inform the interpretation of network structures. They develop the notion of ‘circuits of education’ to show how structures in the network emerge from the repeated spatial interaction of students with a similar background.

Second, we want to highlight how various contributions to the special issue combine network abstraction and contextualization. As Derudder (2021 [this issue]) argues, what the network will show is dependent on those aspects that the researcher designated as nodes or edges. Hall and Penny's (2020 [this issue]) article reports on extraordinary efforts to use palaeoenvironmental data to identify the constituents of a network, but finding that data, constructing the network and interpreting the network structure all require profound domain knowledge. Similarly, although Nic Lochlainn's (2021 [this issue]) analysis treats Facebook links as edges to build ‘an initial and publicly accessible picture of connections between and across online social movement networks’ but then goes on to use digital ethnography and additional interviews to better understand how contention is grounded in places and networked across space.

These seven papers are complemented by two shorter commentaries that evaluate the propositions behind the theory and practice of geographical network analysis. Beate Volker (2021, this issue) compares the efforts of geography with those of sociology to come to terms with network analysis in the computational social science moment, weighing each social science discipline's merits. She questions whether defining a specifically geographical approach to network analysis is beneficial to the interdisciplinary project of network analysis. Gibadullina *et al*. (2021, this issue) push geographical network analysis even further in the geographical tradition. They show how methodological insights from (critical) GIS and experimental spatial mathematics could strengthen the dialogue with both computational approaches and post‐structuralist geography.

## CONCLUSION

In the 1960s, geographers were at the forefront of what is today known as ‘network analysis’. By using networks to operationalize a wide range of natural and social phenomena – ranging from river deltas and migration flows to transport infrastructures and trade relations – quantitative geographers of the 1960s attempted to develop a shared vocabulary to identify universal patterns and develop parsimonious explanations. Although this kind of scientific enterprise fell out of favour within geography, it remains prominent in other disciplines. Complexity scientists, in particular, breach the divide between the social and natural sciences and use network analysis as a common vocabulary to analyse across a wide range of domains.

We argued that, for practical as well as principled reasons, geography as a discipline cannot and should not reclaim its position as front‐runner in the creation of frameworks and methods designed to identify universal patterns and develop parsimonious explanations. We say that despite the valuable and laudable contributions of individual scholars and subcommunities of geographers. These scholars do not just contribute to the research frontiers in network analysis and computational social science, but more importantly, provide the bridge that make insights from geography have a seat at the table of this important research frontier. But what should geography as a discipline do? What does or should geographical network analysis look like? With the exception of some specific bodies of work – notably urban networks research and research on regional economic agglomeration – the adoption of network analysis in geography has so far been rather diffused, uneven and ad hoc. The result has been that many researchers have incidentally used network analysis for specific purposes but that broader debates across subdisciplines – specifically about epistemology – have not been taking place.

In this context, we identified a number of key precepts for geographical network analysis. We suggest that geographers are well positioned to develop strands of network analysis that build on their disciplinary strengths, including domain knowledge, a theoretical understanding of complex spatialities, an appreciation of particularitiesand a commitment to mixed methods. Building on these strengths may complicate the search for invariant laws or hidden order. However, the historical trajectory of the discipline shows us that this complication is a contribution rather than a liability in interdisciplinary debates. Geography helps understand the uneven development of various kinds of networks across space. Geography also has the vocabulary to criticize and qualify the hidden order and to identify the left behind and those likely to be overlooked in the analysis. As network analysts in other disciplines increasingly acknowledge the merits of a spatial perspective, we hope that geographers build on their disciplinary strengths to use network analysis to make their distinctive mark.

## Notes

^1^
There are many strands within computational social science (e.g. Edelmann et al., 2020). Nevertheless, foundational statements on computational social science propose to examine social life through the prism of complexity (e.g. Conte et al., 2012).

^2^
Haggett and Chorley (1969) and Tinkler (1979) provide more comprehensive overviews of the network studies conducted in geography in this era.
